# Diethyl 2-*tert*-butyl-4,11-dioxo-2,3-di­hydro-*cis*-1*H*,5*H*,10*H*-2,3a,4a,10a,11a-penta­azabenzo[*f*]indeno[2,1,7-*ija*]azulene-11b,11c-dicarboxyl­ate

**DOI:** 10.1107/S1600536810000097

**Published:** 2010-01-09

**Authors:** Yanping Zhu, Yichong Sun, Mingqiang Qiu

**Affiliations:** a Key Laboratory of Pesticides and Chemical Biology of the Ministry of Education, College of Chemistry, Central China Normal University, Wuhan 430079, People’s Republic of China

## Abstract

In the title mol­ecule, C_24_H_31_N_5_O_6_, the two ethyl fragments are each disordered over two conformations [occupancy ratios 0.58 (13)/0.42 (13) and 0.56 (12)/0.44 (12)]. The crystal packing exhibits inter­molecular non-classical C—H⋯O hydrogen bonds and π–π inter­actions between benzene rings [centroid–centroid distances = 3.836 (5) Å].

## Related literature

For the preparation of the title compound, see: Wu *et al.* (2002 ▶). For crystal engineering studies on glycoluril and its derivatives, see Chen *et al.* (2007 ▶); Wang *et al.* (2006 ▶).
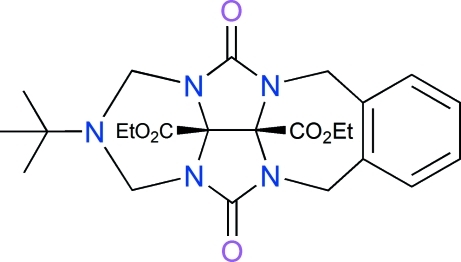


## Experimental

### 

#### Crystal data

C_24_H_31_N_5_O_6_
*M* *_r_* = 485.54
Triclinic, 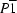

*a* = 10.7133 (14) Å
*b* = 11.1013 (15) Å
*c* = 11.3352 (15) Å
α = 100.493 (2)°
β = 105.438 (2)°
γ = 102.445 (2)°
*V* = 1227.2 (3) Å^3^
*Z* = 2
Mo *K*α radiation
μ = 0.10 mm^−1^
*T* = 292 K
0.30 × 0.20 × 0.20 mm

#### Data collection

Bruker SMART 4K CCD area-detector diffractometer
9853 measured reflections
4253 independent reflections
2734 reflections with *I* > 2σ(*I*)
*R*_int_ = 0.051

#### Refinement

*R*[*F* ^2^ > 2σ(*F* ^2^)] = 0.062
*wR*(*F* ^2^) = 0.195
*S* = 1.03
4253 reflections
397 parameters
99 restraints
H-atom parameters constrained
Δρ_max_ = 0.46 e Å^−3^
Δρ_min_ = −0.27 e Å^−3^

### 

Data collection: *SMART* (Bruker, 1997 ▶); cell refinement: *SAINT* (Bruker, 1999 ▶); data reduction: *SAINT*; program(s) used to solve structure: *SHELXS97* (Sheldrick, 2008 ▶); program(s) used to refine structure: *SHELXL97* (Sheldrick, 2008 ▶); molecular graphics: *SHELXTL* (Sheldrick, 2008 ▶); software used to prepare material for publication: *SHELXTL*.

## Supplementary Material

Crystal structure: contains datablocks I, global. DOI: 10.1107/S1600536810000097/cv2681sup1.cif

Structure factors: contains datablocks I. DOI: 10.1107/S1600536810000097/cv2681Isup2.hkl

Additional supplementary materials:  crystallographic information; 3D view; checkCIF report

## Figures and Tables

**Table 1:** Hydrogen-bond geometry (Å, °)

*D*—H⋯*A*	*D*—H	H⋯*A*	*D*⋯*A*	*D*—H⋯*A*
C22—H22*C*⋯O5^i^	0.96	2.52	3.474 (5)	174
C13—H13*A*⋯O1^ii^	0.97	2.54	3.288 (11)	134

Symmetry codes: (i) 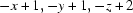; (ii) 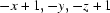.

**Fig. 1.**
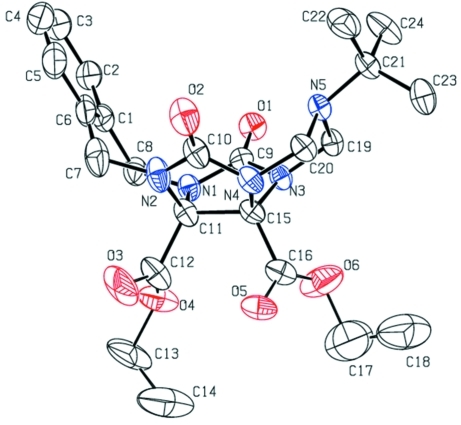
A view of (I), showing the atom-labelling scheme, with displacement ellipsoids drawn at the 30% probability level. Only major parts of disordered atoms are shown. H atoms omitted for clarity.

## supplementary crystallographic information

### Comment

As a continuation of our structural studies of glucoluril derivatives (Wang *et al.*, 2006; Chen *et al.*, 2007), herein we report the crystal structure of the title compound, (I) (Fig. 1). In the title compound, a benzene ring is fused to one seven-membered ring, which binds two of the N atoms from separate rings of the glycoluril system, and the other two N atoms are linked to *tert*-butylamine by two methylene group.

### Experimental

The title compound was synthesized according to the reported literature (Wu *et al.*, 2002). Crystals of (I) suitable for X-ray diffraction were grown by slow evaporation of a dichloromethane-methanol (1:2) solution of the title compound under 293 K.

### Refinement

All H-atoms were positioned geometrically in idealized positions and constrained to ride on their parent atoms, with d(C—H) = 0.97 Å, *U*_iso_ = 1.2*U*_eq_ (C) for CH_2_ and d(C—H) = 0.96 Å, *U*_iso_ = 1.5*U*_eq_ (C) for CH_3_ atoms. The two ethyl group were treated as disordered over two positions.The occupancies of the disordered positions refined to 0.58 (13)/0.42 (13) and 0.56 (12)/0.44 (12), respectively.

### Crystal data

**Table:**

C_24_H_31_N_5_O_6_	*Z* = 2
*M**_r_* = 485.54	*F*(000) = 516
Triclinic, *P*1	*D*_x_ = 1.314 Mg m^−^^3^
*a* = 10.7133 (14) Å	Mo *K*α radiation, λ = 0.71073 Å
*b* = 11.1013 (15) Å	Cell parameters from 2728 reflections
*c* = 11.3352 (15) Å	θ = 2.3–23.3°
α = 100.493 (2)°	µ = 0.10 mm^−^^1^
β = 105.438 (2)°	*T* = 292 K
γ = 102.445 (2)°	Block, colourless
*V* = 1227.2 (3) Å^3^	0.30 × 0.20 × 0.20 mm

### Data collection

**Table:**

Bruker SMART 4K CCD area-detector diffractometer	2734 reflections with *I* > 2σ(*I*)
Radiation source: fine-focus sealed tube	*R*_int_ = 0.051
graphite	θ_max_ = 25.0°, θ_min_ = 1.9°
phi and ω scans	*h* = −12→12
9853 measured reflections	*k* = −13→13
4253 independent reflections	*l* = −13→13

### Refinement

**Table:**

Refinement on *F*^2^	Primary atom site location: structure-invariant direct methods
Least-squares matrix: full	Secondary atom site location: difference Fourier map
*R*[*F*^2^ > 2σ(*F*^2^)] = 0.062	Hydrogen site location: inferred from neighbouring sites
*wR*(*F*^2^) = 0.195	H-atom parameters constrained
*S* = 1.02	*w* = 1/[σ^2^(*F*_o_^2^) + (0.116*P*)^2^] where *P* = (*F*_o_^2^ + 2*F*_c_^2^)/3
4253 reflections	(Δ/σ)_max_ < 0.001
397 parameters	Δρ_max_ = 0.46 e Å^−^^3^
99 restraints	Δρ_min_ = −0.27 e Å^−^^3^

### Special details

**Table:**

Geometry. All e.s.d.'s (except the e.s.d. in the dihedral angle between two l.s. planes) are estimated using the full covariance matrix. The cell e.s.d.'s are taken into account individually in the estimation of e.s.d.'s in distances, angles and torsion angles; correlations between e.s.d.'s in cell parameters are only used when they are defined by crystal symmetry. An approximate (isotropic) treatment of cell e.s.d.'s is used for estimating e.s.d.'s involving l.s. planes.
Refinement. Refinement of *F*^2^ against ALL reflections. The weighted *R*-factor *wR* and goodness of fit *S* are based on *F*^2^, conventional *R*-factors *R* are based on *F*, with *F* set to zero for negative *F*^2^. The threshold expression of *F*^2^ > σ(*F*^2^) is used only for calculating *R*-factors(gt) *etc*. and is not relevant to the choice of reflections for refinement. *R*-factors based on *F*^2^ are statistically about twice as large as those based on *F*, and *R*- factors based on ALL data will be even larger.

### Fractional atomic coordinates and isotropic or equivalent isotropic displacement parameters (Å^2^)

**Table:**

	*x*	*y*	*z*	*U*_iso_*/*U*_eq_	Occ. (<1)
C1	0.2330 (3)	−0.0629 (3)	0.5732 (4)	0.0707 (9)	
C2	0.1387 (4)	−0.1323 (3)	0.4603 (5)	0.0946 (12)	
H2	0.1646	−0.1474	0.3881	0.114*	
C3	0.0032 (5)	−0.1810 (4)	0.4517 (7)	0.131 (2)	
H3	−0.0605	−0.2276	0.3745	0.157*	
C4	−0.0332 (6)	−0.1590 (6)	0.5575 (10)	0.141 (3)	
H4	−0.1225	−0.1915	0.5525	0.169*	
C5	0.0599 (6)	−0.0892 (5)	0.6724 (7)	0.1203 (18)	
H5	0.0326	−0.0752	0.7439	0.144*	
C6	0.1936 (4)	−0.0398 (3)	0.6825 (4)	0.0825 (11)	
C7	0.2936 (5)	0.0298 (3)	0.8120 (4)	0.0916 (12)	
H7A	0.2447	0.0388	0.8723	0.110*	
H7B	0.3532	−0.0220	0.8374	0.110*	
C8	0.3760 (3)	−0.0180 (2)	0.5746 (3)	0.0634 (8)	
H8A	0.4280	−0.0669	0.6193	0.076*	
H8B	0.3772	−0.0372	0.4880	0.076*	
C9	0.4226 (3)	0.2080 (2)	0.5675 (2)	0.0450 (6)	
C10	0.3472 (3)	0.2665 (3)	0.8592 (3)	0.0641 (8)	
C11	0.4844 (3)	0.1701 (2)	0.7674 (2)	0.0576 (7)	
C12	0.5931 (4)	0.1179 (3)	0.8426 (3)	0.0844 (11)	
C13	0.7589 (10)	0.0161 (11)	0.8247 (10)	0.138 (4)	0.58 (13)
H13A	0.7778	−0.0399	0.7594	0.166*	0.58
H13B	0.7242	−0.0352	0.8757	0.166*	0.58
C14	0.8840 (12)	0.1122 (12)	0.9046 (13)	0.184 (6)	0.58 (13)
H14A	0.9079	0.1746	0.8599	0.276*	0.58
H14B	0.9548	0.0719	0.9253	0.276*	0.58
H14C	0.8712	0.1533	0.9809	0.276*	0.58
C15	0.5263 (3)	0.3179 (2)	0.7817 (2)	0.0504 (7)	
C16	0.6759 (4)	0.3861 (3)	0.8485 (3)	0.0730 (9)	
C17	0.8800 (10)	0.4862 (14)	0.8238 (14)	0.188 (5)	0.56 (12)
H17A	0.9208	0.4584	0.8974	0.225*	0.56
H17B	0.9202	0.4634	0.7588	0.225*	0.56
C18	0.8945 (16)	0.6226 (16)	0.856 (2)	0.190 (8)	0.56
H18A	0.8694	0.6505	0.7802	0.286*	0.56
H18B	0.9865	0.6674	0.9046	0.286*	0.56
H18C	0.8370	0.6399	0.9055	0.286*	0.56
C19	0.4409 (3)	0.4385 (2)	0.6278 (2)	0.0519 (7)	
H19A	0.3984	0.4254	0.5377	0.062*	
H19B	0.5196	0.5119	0.6559	0.062*	
C20	0.4129 (3)	0.4808 (2)	0.8311 (2)	0.0569 (7)	
H20A	0.4942	0.5514	0.8604	0.068*	
H20B	0.3532	0.5012	0.8779	0.068*	
C21	0.2878 (3)	0.5689 (3)	0.6669 (3)	0.0642 (8)	
C22	0.1716 (4)	0.5641 (4)	0.7200 (4)	0.0937 (11)	
H22A	0.1086	0.4808	0.6865	0.141*	
H22B	0.1272	0.6267	0.6965	0.141*	
H22C	0.2053	0.5818	0.8106	0.141*	
C23	0.3910 (4)	0.7005 (3)	0.7234 (4)	0.0996 (12)	
H23A	0.4286	0.7134	0.8132	0.149*	
H23B	0.3472	0.7654	0.7068	0.149*	
H23C	0.4616	0.7051	0.6857	0.149*	
C24	0.2300 (4)	0.5452 (4)	0.5232 (3)	0.0966 (12)	
H24A	0.3019	0.5505	0.4871	0.145*	
H24B	0.1848	0.6085	0.5040	0.145*	
H24C	0.1671	0.4620	0.4885	0.145*	
C13'	0.8049 (12)	0.0858 (17)	0.867 (2)	0.169 (8)	0.42 (13)
H13C	0.8264	0.1281	0.9558	0.203*	0.42
H13D	0.8837	0.1142	0.8419	0.203*	0.42
C14'	0.7753 (15)	−0.0522 (15)	0.8527 (12)	0.165 (6)	0.42 (13)
H14D	0.7121	−0.0780	0.8957	0.248*	0.42
H14E	0.8571	−0.0735	0.8885	0.248*	0.42
H14F	0.7374	−0.0957	0.7645	0.248*	0.42
C17'	0.8558 (11)	0.5710 (11)	0.8799 (13)	0.095 (3)	0.44 (12)
H17C	0.9176	0.5259	0.8575	0.113*	0.44
H17D	0.8793	0.5963	0.9713	0.113*	0.44
C18'	0.8578 (12)	0.6842 (12)	0.8253 (13)	0.127 (5)	0.44 (12)
H18D	0.8380	0.6571	0.7353	0.190*	0.44
H18E	0.9454	0.7444	0.8619	0.190*	0.44
H18F	0.7913	0.7236	0.8435	0.190*	0.44
N1	0.4438 (2)	0.11805 (18)	0.63279 (19)	0.0510 (6)	
N2	0.3746 (3)	0.1548 (2)	0.8183 (2)	0.0725 (8)	
N3	0.4818 (2)	0.32668 (18)	0.65159 (18)	0.0479 (5)	
N4	0.4467 (2)	0.3659 (2)	0.85387 (19)	0.0550 (6)	
N5	0.3465 (2)	0.46257 (18)	0.69526 (18)	0.0503 (6)	
O1	0.36756 (19)	0.18819 (16)	0.45526 (17)	0.0599 (5)	
O2	0.2539 (3)	0.2773 (2)	0.8978 (2)	0.0899 (8)	
O3	0.5872 (17)	0.0875 (14)	0.9359 (8)	0.135 (5)	0.58 (13)
O4	0.6596 (7)	0.0723 (8)	0.7670 (7)	0.098 (3)	0.58 (13)
O5	0.7302 (5)	0.3777 (6)	0.9524 (3)	0.0774 (14)	0.56 (12)
O6	0.7316 (6)	0.4306 (9)	0.7765 (6)	0.136 (3)	0.56
O3'	0.624 (2)	0.1280 (19)	0.9535 (9)	0.124 (6)	0.42 (13)
O4'	0.6965 (9)	0.1227 (10)	0.7948 (10)	0.086 (3)	0.42 (13)
O5'	0.7607 (13)	0.3412 (14)	0.893 (2)	0.259 (8)	0.44 (12)
O6'	0.7172 (6)	0.4933 (5)	0.8223 (8)	0.091 (3)	0.44 (12)

### Atomic displacement parameters (Å^2^)

**Table:**

	*U*^11^	*U*^22^	*U*^33^	*U*^12^	*U*^13^	*U*^23^
C1	0.068 (2)	0.0460 (16)	0.113 (3)	0.0248 (15)	0.034 (2)	0.0346 (17)
C2	0.074 (3)	0.0547 (19)	0.145 (4)	0.0146 (18)	0.015 (3)	0.033 (2)
C3	0.086 (4)	0.074 (3)	0.220 (7)	0.019 (2)	0.015 (4)	0.060 (4)
C4	0.075 (3)	0.089 (3)	0.287 (9)	0.031 (3)	0.067 (5)	0.092 (5)
C5	0.102 (4)	0.084 (3)	0.235 (6)	0.047 (3)	0.100 (4)	0.089 (4)
C6	0.081 (2)	0.0564 (18)	0.151 (4)	0.0345 (18)	0.068 (3)	0.059 (2)
C7	0.130 (3)	0.073 (2)	0.116 (3)	0.041 (2)	0.082 (3)	0.051 (2)
C8	0.069 (2)	0.0480 (15)	0.082 (2)	0.0226 (14)	0.0330 (16)	0.0182 (14)
C9	0.0465 (15)	0.0489 (14)	0.0454 (15)	0.0169 (11)	0.0220 (12)	0.0110 (12)
C10	0.094 (2)	0.0698 (18)	0.0530 (17)	0.0396 (17)	0.0424 (17)	0.0273 (14)
C11	0.076 (2)	0.0577 (15)	0.0579 (18)	0.0337 (14)	0.0313 (15)	0.0261 (13)
C12	0.119 (3)	0.083 (2)	0.068 (2)	0.054 (2)	0.026 (2)	0.035 (2)
C13	0.142 (7)	0.122 (7)	0.149 (7)	0.104 (6)	0.001 (5)	0.015 (6)
C14	0.126 (9)	0.213 (12)	0.166 (10)	0.093 (9)	−0.029 (8)	−0.008 (8)
C15	0.0614 (18)	0.0584 (15)	0.0383 (14)	0.0277 (13)	0.0170 (13)	0.0143 (12)
C16	0.070 (2)	0.082 (2)	0.071 (2)	0.0379 (19)	0.0151 (19)	0.0211 (18)
C17	0.133 (8)	0.218 (10)	0.195 (9)	0.029 (7)	0.039 (7)	0.052 (8)
C18	0.122 (13)	0.208 (17)	0.178 (15)	0.043 (12)	−0.013 (10)	−0.010 (12)
C19	0.0669 (18)	0.0457 (13)	0.0460 (15)	0.0161 (12)	0.0200 (13)	0.0153 (11)
C20	0.0750 (19)	0.0546 (15)	0.0469 (16)	0.0286 (14)	0.0211 (14)	0.0119 (12)
C21	0.077 (2)	0.0558 (16)	0.0696 (19)	0.0343 (15)	0.0210 (16)	0.0244 (14)
C22	0.097 (3)	0.101 (3)	0.112 (3)	0.060 (2)	0.044 (2)	0.040 (2)
C23	0.121 (3)	0.0509 (18)	0.133 (3)	0.0342 (19)	0.037 (3)	0.0299 (19)
C24	0.128 (3)	0.106 (3)	0.073 (2)	0.072 (3)	0.018 (2)	0.038 (2)
C13'	0.144 (12)	0.169 (12)	0.190 (13)	0.068 (9)	0.028 (9)	0.047 (9)
C14'	0.185 (12)	0.174 (12)	0.103 (8)	0.125 (11)	−0.024 (8)	−0.029 (8)
C17'	0.053 (6)	0.077 (6)	0.121 (7)	−0.002 (4)	0.016 (5)	−0.013 (5)
C18'	0.093 (8)	0.138 (9)	0.100 (7)	−0.040 (7)	−0.001 (7)	0.043 (7)
N1	0.0648 (14)	0.0454 (11)	0.0523 (13)	0.0213 (10)	0.0270 (11)	0.0156 (10)
N2	0.108 (2)	0.0633 (14)	0.0836 (17)	0.0394 (14)	0.0650 (16)	0.0379 (13)
N3	0.0620 (14)	0.0450 (12)	0.0406 (12)	0.0193 (10)	0.0175 (10)	0.0132 (10)
N4	0.0752 (16)	0.0602 (13)	0.0400 (12)	0.0315 (12)	0.0226 (11)	0.0169 (10)
N5	0.0635 (14)	0.0456 (11)	0.0454 (12)	0.0202 (10)	0.0181 (11)	0.0133 (9)
O1	0.0706 (13)	0.0613 (11)	0.0430 (11)	0.0178 (9)	0.0145 (10)	0.0078 (9)
O2	0.122 (2)	0.0931 (16)	0.1017 (17)	0.0523 (14)	0.0815 (16)	0.0441 (13)
O3	0.172 (10)	0.171 (9)	0.107 (7)	0.076 (7)	0.047 (6)	0.111 (7)
O4	0.086 (4)	0.109 (5)	0.080 (4)	0.054 (4)	−0.009 (3)	−0.002 (3)
O5	0.072 (3)	0.115 (4)	0.0313 (19)	0.032 (3)	−0.0084 (19)	0.015 (2)
O6	0.070 (4)	0.231 (7)	0.089 (4)	−0.007 (5)	0.016 (3)	0.069 (5)
O3'	0.141 (11)	0.172 (12)	0.069 (6)	0.092 (10)	0.012 (5)	0.028 (5)
O4'	0.075 (5)	0.108 (7)	0.082 (6)	0.057 (5)	0.013 (5)	0.018 (5)
O5'	0.153 (10)	0.165 (11)	0.417 (19)	0.032 (8)	−0.001 (11)	0.124 (13)
O6'	0.054 (4)	0.059 (3)	0.137 (7)	0.008 (3)	0.012 (4)	0.008 (3)

### Geometric parameters (Å, °)

**Table:**

C1—C2	1.367 (5)	C16—O6	1.243 (6)
C1—C6	1.410 (5)	C16—O6'	1.293 (6)
C1—C8	1.500 (4)	C17—C18	1.455 (17)
C2—C3	1.406 (7)	C17—O6	1.483 (10)
C2—H2	0.9300	C17—H17A	0.9700
C3—C4	1.354 (8)	C17—H17B	0.9700
C3—H3	0.9300	C18—H18A	0.9600
C4—C5	1.378 (8)	C18—H18B	0.9600
C4—H4	0.9300	C18—H18C	0.9600
C5—C6	1.385 (6)	C19—N3	1.449 (3)
C5—H5	0.9300	C19—N5	1.460 (3)
C6—C7	1.514 (5)	C19—H19A	0.9700
C7—N2	1.448 (4)	C19—H19B	0.9700
C7—H7A	0.9700	C20—N4	1.447 (3)
C7—H7B	0.9700	C20—N5	1.469 (3)
C8—N1	1.468 (3)	C20—H20A	0.9700
C8—H8A	0.9700	C20—H20B	0.9700
C8—H8B	0.9700	C21—N5	1.498 (3)
C9—O1	1.207 (3)	C21—C22	1.516 (4)
C9—N1	1.369 (3)	C21—C24	1.530 (4)
C9—N3	1.381 (3)	C21—C23	1.534 (5)
C10—O2	1.212 (3)	C22—H22A	0.9600
C10—N2	1.363 (4)	C22—H22B	0.9600
C10—N4	1.382 (4)	C22—H22C	0.9600
C11—N2	1.434 (3)	C23—H23A	0.9600
C11—N1	1.439 (3)	C23—H23B	0.9600
C11—C12	1.536 (4)	C23—H23C	0.9600
C11—C15	1.571 (4)	C24—H24A	0.9600
C12—O3	1.180 (7)	C24—H24B	0.9600
C12—O3'	1.189 (9)	C24—H24C	0.9600
C12—O4	1.350 (6)	C13'—O4'	1.418 (8)
C12—O4'	1.352 (7)	C13'—C14'	1.465 (14)
C13—O4	1.418 (7)	C13'—H13C	0.9700
C13—C14	1.463 (12)	C13'—H13D	0.9700
C13—H13A	0.9700	C14'—H14D	0.9600
C13—H13B	0.9700	C14'—H14E	0.9600
C14—H14A	0.9600	C14'—H14F	0.9600
C14—H14B	0.9600	C17'—O6'	1.450 (10)
C14—H14C	0.9600	C17'—C18'	1.497 (17)
C15—N4	1.445 (3)	C17'—H17C	0.9700
C15—N3	1.452 (3)	C17'—H17D	0.9700
C15—C16	1.529 (4)	C18'—H18D	0.9600
C16—O5'	1.170 (9)	C18'—H18E	0.9600
C16—O5	1.197 (4)	C18'—H18F	0.9600
			
C2—C1—C6	119.5 (3)	N3—C19—N5	109.90 (18)
C2—C1—C8	117.4 (3)	N3—C19—H19A	109.7
C6—C1—C8	123.1 (3)	N5—C19—H19A	109.7
C1—C2—C3	120.9 (5)	N3—C19—H19B	109.7
C1—C2—H2	119.5	N5—C19—H19B	109.7
C3—C2—H2	119.5	H19A—C19—H19B	108.2
C4—C3—C2	119.0 (6)	N4—C20—N5	110.0 (2)
C4—C3—H3	120.5	N4—C20—H20A	109.7
C2—C3—H3	120.5	N5—C20—H20A	109.7
C3—C4—C5	121.2 (5)	N4—C20—H20B	109.7
C3—C4—H4	119.4	N5—C20—H20B	109.7
C5—C4—H4	119.4	H20A—C20—H20B	108.2
C4—C5—C6	120.6 (5)	N5—C21—C22	107.9 (2)
C4—C5—H5	119.7	N5—C21—C24	108.7 (2)
C6—C5—H5	119.7	C22—C21—C24	107.6 (3)
C5—C6—C1	118.8 (5)	N5—C21—C23	112.8 (2)
C5—C6—C7	118.7 (4)	C22—C21—C23	109.6 (3)
C1—C6—C7	122.4 (3)	C24—C21—C23	110.1 (3)
N2—C7—C6	114.3 (3)	C21—C22—H22A	109.5
N2—C7—H7A	108.7	C21—C22—H22B	109.5
C6—C7—H7A	108.7	H22A—C22—H22B	109.5
N2—C7—H7B	108.7	C21—C22—H22C	109.5
C6—C7—H7B	108.7	H22A—C22—H22C	109.5
H7A—C7—H7B	107.6	H22B—C22—H22C	109.5
N1—C8—C1	116.0 (2)	C21—C23—H23A	109.5
N1—C8—H8A	108.3	C21—C23—H23B	109.5
C1—C8—H8A	108.3	H23A—C23—H23B	109.5
N1—C8—H8B	108.3	C21—C23—H23C	109.5
C1—C8—H8B	108.3	H23A—C23—H23C	109.5
H8A—C8—H8B	107.4	H23B—C23—H23C	109.5
O1—C9—N1	126.4 (2)	C21—C24—H24A	109.5
O1—C9—N3	125.6 (2)	C21—C24—H24B	109.5
N1—C9—N3	107.9 (2)	H24A—C24—H24B	109.5
O2—C10—N2	125.9 (3)	C21—C24—H24C	109.5
O2—C10—N4	125.6 (3)	H24A—C24—H24C	109.5
N2—C10—N4	108.5 (2)	H24B—C24—H24C	109.5
N2—C11—N1	113.7 (2)	O4'—C13'—C14'	113.2 (14)
N2—C11—C12	108.8 (2)	O4'—C13'—H13C	108.9
N1—C11—C12	113.5 (2)	C14'—C13'—H13C	108.9
N2—C11—C15	102.01 (19)	O4'—C13'—H13D	108.9
N1—C11—C15	102.94 (17)	C14'—C13'—H13D	108.9
C12—C11—C15	115.4 (3)	H13C—C13'—H13D	107.8
O3—C12—O3'	24.2 (17)	C13'—C14'—H14D	109.5
O3—C12—O4	124.6 (9)	C13'—C14'—H14E	109.5
O3'—C12—O4	124.5 (10)	H14D—C14'—H14E	109.5
O3—C12—O4'	126.2 (9)	C13'—C14'—H14F	109.5
O3'—C12—O4'	114.3 (12)	H14D—C14'—H14F	109.5
O4—C12—O4'	25.0 (6)	H14E—C14'—H14F	109.5
O3—C12—C11	121.6 (8)	O6'—C17'—C18'	103.2 (9)
O3'—C12—C11	125.3 (10)	O6'—C17'—H17C	111.1
O4—C12—C11	109.8 (4)	C18'—C17'—H17C	111.1
O4'—C12—C11	111.9 (5)	O6'—C17'—H17D	111.1
O4—C13—C14	111.9 (9)	C18'—C17'—H17D	111.1
O4—C13—H13A	109.2	H17C—C17'—H17D	109.1
C14—C13—H13A	109.2	C17'—C18'—H18D	109.5
O4—C13—H13B	109.2	C17'—C18'—H18E	109.5
C14—C13—H13B	109.2	H18D—C18'—H18E	109.5
H13A—C13—H13B	107.9	C17'—C18'—H18F	109.5
N4—C15—N3	111.76 (19)	H18D—C18'—H18F	109.5
N4—C15—C16	109.9 (2)	H18E—C18'—H18F	109.5
N3—C15—C16	112.5 (2)	C9—N1—C11	111.99 (19)
N4—C15—C11	104.0 (2)	C9—N1—C8	121.2 (2)
N3—C15—C11	102.7 (2)	C11—N1—C8	121.5 (2)
C16—C15—C11	115.6 (2)	C10—N2—C11	113.5 (2)
O5'—C16—O5	44.5 (12)	C10—N2—C7	124.6 (3)
O5'—C16—O6	95.8 (12)	C11—N2—C7	121.6 (2)
O5—C16—O6	126.7 (5)	C9—N3—C19	122.0 (2)
O5'—C16—O6'	115.4 (9)	C9—N3—C15	111.98 (19)
O5—C16—O6'	116.8 (5)	C19—N3—C15	118.26 (19)
O6—C16—O6'	39.5 (5)	C10—N4—C15	110.4 (2)
O5'—C16—C15	127.3 (8)	C10—N4—C20	118.8 (2)
O5—C16—C15	120.3 (4)	C15—N4—C20	115.72 (19)
O6—C16—C15	112.1 (4)	C19—N5—C20	108.5 (2)
O6'—C16—C15	114.4 (4)	C19—N5—C21	114.05 (19)
C18—C17—O6	102.7 (10)	C20—N5—C21	112.94 (19)
C18—C17—H17A	111.2	C12—O4—C13	115.0 (7)
O6—C17—H17A	111.2	C16—O6—C17	118.2 (7)
C18—C17—H17B	111.2	C12—O4'—C13'	114.1 (9)
O6—C17—H17B	111.2	C16—O6'—C17'	121.3 (7)
H17A—C17—H17B	109.1		
			
C6—C1—C2—C3	−0.1 (4)	N4—C10—N2—C7	179.1 (3)
C8—C1—C2—C3	178.2 (3)	N1—C11—N2—C10	−110.4 (3)
C1—C2—C3—C4	−0.4 (6)	C12—C11—N2—C10	122.1 (3)
C2—C3—C4—C5	0.5 (7)	C15—C11—N2—C10	−0.3 (3)
C3—C4—C5—C6	0.0 (6)	N1—C11—N2—C7	63.1 (3)
C4—C5—C6—C1	−0.5 (5)	C12—C11—N2—C7	−64.4 (4)
C4—C5—C6—C7	−176.6 (3)	C15—C11—N2—C7	173.2 (3)
C2—C1—C6—C5	0.5 (4)	C6—C7—N2—C10	97.0 (4)
C8—C1—C6—C5	−177.6 (3)	C6—C7—N2—C11	−75.7 (4)
C2—C1—C6—C7	176.5 (3)	O1—C9—N3—C19	25.1 (4)
C8—C1—C6—C7	−1.7 (4)	N1—C9—N3—C19	−158.1 (2)
C5—C6—C7—N2	−128.4 (3)	O1—C9—N3—C15	173.8 (2)
C1—C6—C7—N2	55.6 (4)	N1—C9—N3—C15	−9.4 (3)
C2—C1—C8—N1	129.5 (3)	N5—C19—N3—C9	98.0 (2)
C6—C1—C8—N1	−52.3 (3)	N5—C19—N3—C15	−48.9 (3)
N2—C11—C12—O3	−8.9 (10)	N4—C15—N3—C9	−111.2 (2)
N1—C11—C12—O3	−136.5 (9)	C16—C15—N3—C9	124.6 (2)
C15—C11—C12—O3	105.0 (10)	C11—C15—N3—C9	−0.3 (3)
N2—C11—C12—O3'	−37.6 (14)	N4—C15—N3—C19	38.8 (3)
N1—C11—C12—O3'	−165.2 (14)	C16—C15—N3—C19	−85.3 (3)
C15—C11—C12—O3'	76.3 (14)	C11—C15—N3—C19	149.8 (2)
N2—C11—C12—O4	149.7 (5)	O2—C10—N4—C15	−169.4 (3)
N1—C11—C12—O4	22.0 (5)	N2—C10—N4—C15	12.9 (3)
C15—C11—C12—O4	−96.4 (5)	O2—C10—N4—C20	−32.3 (4)
N2—C11—C12—O4'	176.4 (6)	N2—C10—N4—C20	150.0 (2)
N1—C11—C12—O4'	48.7 (6)	N3—C15—N4—C10	97.5 (2)
C15—C11—C12—O4'	−69.7 (6)	C16—C15—N4—C10	−136.9 (2)
N2—C11—C15—N4	7.6 (3)	C11—C15—N4—C10	−12.6 (3)
N1—C11—C15—N4	125.7 (2)	N3—C15—N4—C20	−41.0 (3)
C12—C11—C15—N4	−110.1 (3)	C16—C15—N4—C20	84.6 (3)
N2—C11—C15—N3	−109.0 (2)	C11—C15—N4—C20	−151.1 (2)
N1—C11—C15—N3	9.1 (2)	N5—C20—N4—C10	−80.5 (3)
C12—C11—C15—N3	133.2 (2)	N5—C20—N4—C15	54.3 (3)
N2—C11—C15—C16	128.1 (2)	N3—C19—N5—C20	58.7 (3)
N1—C11—C15—C16	−113.8 (2)	N3—C19—N5—C21	−174.4 (2)
C12—C11—C15—C16	10.4 (3)	N4—C20—N5—C19	−62.2 (3)
N4—C15—C16—O5'	115.1 (16)	N4—C20—N5—C21	170.3 (2)
N3—C15—C16—O5'	−119.8 (16)	C22—C21—N5—C19	168.0 (3)
C11—C15—C16—O5'	−2.3 (16)	C24—C21—N5—C19	51.7 (3)
N4—C15—C16—O5	61.5 (4)	C23—C21—N5—C19	−70.7 (3)
N3—C15—C16—O5	−173.3 (4)	C22—C21—N5—C20	−67.4 (3)
C11—C15—C16—O5	−55.8 (5)	C24—C21—N5—C20	176.2 (2)
N4—C15—C16—O6	−128.4 (6)	C23—C21—N5—C20	53.8 (3)
N3—C15—C16—O6	−3.2 (6)	O3—C12—O4—C13	−19.6 (14)
C11—C15—C16—O6	114.3 (6)	O3'—C12—O4—C13	9.9 (17)
N4—C15—C16—O6'	−85.3 (5)	O4'—C12—O4—C13	83.1 (19)
N3—C15—C16—O6'	39.9 (5)	C11—C12—O4—C13	−177.3 (7)
C11—C15—C16—O6'	157.4 (5)	C14—C13—O4—C12	−79.4 (12)
O1—C9—N1—C11	−167.1 (2)	O5'—C16—O6—C17	−38.6 (14)
N3—C9—N1—C11	16.2 (3)	O5—C16—O6—C17	−3.6 (13)
O1—C9—N1—C8	−12.6 (4)	O6'—C16—O6—C17	85.1 (10)
N3—C9—N1—C8	170.6 (2)	C15—C16—O6—C17	−173.0 (8)
N2—C11—N1—C9	93.9 (2)	C18—C17—O6—C16	−101.4 (14)
C12—C11—N1—C9	−141.1 (3)	O3—C12—O4'—C13'	0.4 (17)
C15—C11—N1—C9	−15.6 (3)	O3'—C12—O4'—C13'	24.9 (17)
N2—C11—N1—C8	−60.5 (3)	O4—C12—O4'—C13'	−95 (2)
C12—C11—N1—C8	64.5 (3)	C11—C12—O4'—C13'	174.9 (10)
C15—C11—N1—C8	−170.0 (2)	C14'—C13'—O4'—C12	74.8 (19)
C1—C8—N1—C9	−80.3 (3)	O5'—C16—O6'—C17'	−19.1 (18)
C1—C8—N1—C11	71.8 (3)	O5—C16—O6'—C17'	30.7 (11)
O2—C10—N2—C11	174.7 (3)	O6—C16—O6'—C17'	−85.5 (10)
N4—C10—N2—C11	−7.6 (3)	C15—C16—O6'—C17'	178.8 (8)
O2—C10—N2—C7	1.4 (5)	C18'—C17'—O6'—C16	−179.2 (9)

### Hydrogen-bond geometry (Å, °)

**Table:**

*D*—H···*A*	*D*—H	H···*A*	*D*···*A*	*D*—H···*A*
C22—H22C···O5^i^	0.96	2.52	3.474 (5)	174
C13—H13A···O1^ii^	0.97	2.54	3.288 (11)	134

Symmetry codes: (i) −*x*+1, −*y*+1, −*z*+2; (ii) −*x*+1, −*y*, −*z*+1.
